# Morphology vs morphokinetics: a retrospective comparison of inter-observer and intra-observer agreement between embryologists on blastocysts with known implantation outcome

**DOI:** 10.5935/1518-0557.20180042

**Published:** 2018

**Authors:** Emma Adolfsson, Anna Nowosad Andershed

**Affiliations:** 1 Örebro University Hospital. Department of Laboratory Medicine. Örebro, Sweden; 2 Örebro University Hospital. Fertility Unit, Department of Women Health. Örebro, Sweden

**Keywords:** IVF, embryo development, time lapse, morphokinetics, observer agreement

## Abstract

**Objective:**

Our primary aim was to compare the morphology and morphokinetics on inter- and intra-observer agreement for blastocyst with known implantation outcome. Our secondary aim was to validate the morphokinetic parameters' ability to predict pregnancy using a previous published selection algorithm, and to compare this to standard morphology assessments.

**Methods:**

Two embryologists made independent blinded annotations on two occasions using time-lapse images and morphology evaluations using the Gardner Schoolcraft criteria of 99 blastocysts with known implantation outcome. Inter- and intra-observer agreement was calculated and compared using the two methods. The embryos were grouped based on their morphological score, and on their morphokinetic class using a previous published selection algorithm. The implantation rates for each group was calculated and compared.

**Results:**

There was moderate agreement for morphology, with agreement on the same embryo score in 55 of 99 cases. The highest agreement rate was found for expansion grade, followed by trophectoderm and inner cell mass. Correlation with pregnancy was inconclusive. For morphokinetics, almost perfect agreement was found for early and late embryo development events, and strong agreement for day-2 and day-3 events. When applying the selection algorithm, the embryo distributions were uneven, and correlation to pregnancy was inconclusive.

**Conclusions:**

Time-lapse annotation is consistent and accurate, but our external validation of a previously published selection algorithm was unsuccessful.

## INTRODUCTION

Traditionally, scoring and selection of embryos is done through microscopic evaluations of their morphological features. Blastocysts are commonly scored using the Gardner Schoolcraft criteria about the expansion grade, and the number and cohesiveness of cells in the inner cell mass (ICM) and trophectoderm (TE) (Gardner *et al*., 2000). Morphology and developmental competence are not strongly correlated. One of the reasons may be a high degree of inter-observer and intra-observer variability (Arce *et al*., 2006; Baxter Bendus *et al*., 2006; Paternot *et al.,* 2011). This can be explained by the non-rigid definitions of blastocyst grades and the lack of a precise timing for the observations (Alpha/ESHRE, 2011; Montag *et al*., 2011). Because of the relatively low power of morphology to select viable embryos, utilizing time lapse as an embryo selection tool is a tempting alternative. Since 2012, culture in EmbryoScope is the standard practice for all patients attending our Fertility Clinic. The use of time-lapse imaging has truly been a paradigm shift for embryologists. There is available information about embryo changes from a quick morphological observation per embryo per day, to the gathering of approximately 6000 images per embryo during culture. This enables embryologists to play and replay the development, and to evaluate morphological features without exposing the embryos to sub-optimal culture conditions. The increase in information about each embryo should, in theory, increase the likelihood of choosing the embryos with the highest ability to lead to pregnancy.

Describing embryos using time lapse imaging and expressing their development in parameters and patterns of cleavage is called morphokinetics. Morphokinetic parameters date each specific event in embryo development; appearance and fading of pronuclei, each cell stage, cell compaction, morula, blastulation, and blastocyst expansion/herniation/hatching. Numerous studies have investigated the relationship between morphokinetics and embryo competence (Wong *et al*., 2010; Meseguer *et al*., 2011; Azzarello *et al*., 2012; Cruz *et al*., 2012; Dal Canto *et al*., 2012; Hashimoto *et al*., 2012; Hlinka *et al*., 2012) and between morphokinetics and chromosomal content (Basile *et al*., 2014; Campbell *et al.*, 2013, Chawla *et al*., 2015).

Meseguer *et al.* (2011) proposed the first model for embryo selection based on morphokinetic parameters. Their hierarchical classification of cleavage-stage embryos, or flowchart, is based on morphological screening, and then morphokinetics within the cleavage stages. Initially, the embryologist excludes non-viable, arrested or degenerated embryos and assign them the score F. Then, embryos displaying exclusion criteria such as multinucleation at the four-cell stage, uneven blastomere size at the two-cell stage, or direct cleavage are excluded and assigned the score E. Finally, the remaining embryos are ranked based on morphokinetic parameters. First, the time of cell division to five cells (t5) is used. Embryos inside the optimal time interval are scored as A/B, and embryos outside this interval as C/D. Next, a parameter measuring the synchrony of divisions from three to four cells is used. This determines whether the embryo is A or B, or C or D. Finally, the second cell cycle duration, i.e. the time from two to three cells is used to rank the embryos into subgroups, named + or -. Usage of this hierarchical model results in ten embryo classes, from the best score A+ to F.

After the publication of the Meseguer selection (Meseguer *et al*., 2011) model, several other models have been developed, that utilize time-lapse imaging and annotation to select embryos of high quality. A recent meta-analysis confirms the value of morphokinetic embryo selection, as it shows improved pregnancy, higher live birth rates and reduced early pregnancy loss (Pribenszky *et al*., 2017).

For time lapse to replace and/or complement morphology it must be accurate and consistent. For a selection algorithm to be precise, the annotation technique per se needs to be robust, objective and free from bias. Consistency and accuracy are dependent on the variability within and between observers and can be calculated as inter-observer and intra-observer agreement. To our knowledge, only two studies have explored this for annotations so far (Sundvall *et al*., 2013; Martínez-Granados *et al*., 2017). In both studies, the authors concluded that extremely close agreement was found for the majority of investigated parameters, with the remaining parameters indicating close agreement. However, in the study by Sundvall *et al*. (2013), the observations of morphological events at two-cell stage (evenness and multinucleation) showed only fair to moderate agreement. To our knowledge, there has been no study comparing morphology and morphokinetics inter-observer and intra-observer agreement on the same embryo set. Therefore, the aim of this study was to validate and compare morphokinetics to morphology on blastocysts with known implantation outcome, with primary endpoint inter- and intra-observer agreement. Secondary aim was to validate the morphokinetic parameters ability to predict pregnancy using a previous published selection algorithm, and to compare this to standard morphology assessments.

## MATERIAL AND METHODS

### Subjects

The patients attending our Fertility Unit at Örebro University Hospital, Sweden, between 2012-2014 were randomized for participation. The subject sample consisted of patients' embryos with known implantation statuses, which had been transferred as a single day-5 blastocyst from a fresh IVF/ICSI cycle.

### Ethical approval

Written informed consent was obtained from all patients, stating consent towards research and/or methodological development. The project was approved by the local ethics committee (Regionala etikprövningsnämnden Uppsala, ethical approval Ö44-14).

### Ovarian stimulation, oocyte retrieval and ICSI/IVF

Ovarian stimulation and oocyte retrieval was performed as per standard operating procedures. The patients were stimulated using either antagonist (n=67) or agonist protocols (n=32). The ovarian stimulation was carried out by administering recombinant FSH or hMG from cycle days 1-3. Vaginal ultrasound-guided aspiration of oocyte-cumulus complexes was performed 36 hours post-triggering with human chorionic gonadotrophin administration. Following oocyte retrieval, the oocytes were fertilized using standard IVF (n=49) or ICSI (n=50). The IVF embryos were cultured overnight in a conventional incubator using atmospheric oxygen levels, before being placed in EmbryoScope after removal of cumulus cells. ICSI embryos were immediately placed in EmbryoScope after insemination. The EmbryoSlide contained 25 µl of G1 v5 (Vitrolife, Sweden) overlaid with OVOIL (Vitrolife, Sweden) and incubation took place at 37.3ºC, 6% CO_2_ and 5% O_2_. On days 2 and 4 of culture, half-media change was performed by removing 20 µl of old media and replacing it with 25 µl of CCM (Vitrolife, Sweden).

### ET and luteal phase support, pregnancy test and ultrasound

The best embryo - based on strict morphology embryo scoring for blastocysts - was selected for transfer. Elective single embryo transfer was performed on day 5. Luteal phase support was given as per standard operating procedures. A home urine pregnancy test was taken 16 days after embryo transfer. If pregnant, an early vaginal ultrasound was performed after week 6 to confirm a viable pregnancy and number of fetuses.

### Time lapse recording and annotation

Images were recorded every 10 minutes in 7 focal planes over at least 120 hours of culture (15 µm intervals, 1280 x 1024 pixels, 3 pixels per um, monochrome, 8-bit < 0.5s per image using single 1W red LED) and saved at an external work station (EmbryoViewer). t=0 was defined as time of fertilization (for ICSI time of injection, for IVF time of gamete co-incubation). The exact times for each parameter were calculated in hours post insemination (HPI), and the time point is defined as the first frame of observation of the event recorded. Two observers performed annotation manually; both observers were ESHRE certified embryologists with five and ten years of experience of assisted reproduction, respectively. Both observers have several years of experience using EmbryoScope for all patients. Each embryo was scored four times, two times by each examiner. The scoring was performed blindly, i.e. the embryologist was blinded both to previous assessments and to the outcome of the transferred embryo. The annotation was done two months apart in time.

The following parameters were annotated using time points at each morphokinetic scoring; tPNa as the time of appearance of pronuclei, tPNf as the time of pronuclei fading. t2, t3, t4, t5, t6, t7, t8, t9+ was defined as the times for the corresponding number of cells. tM was defined as the first frame of the morula stage, tSB as the first frame with presence of blastocoel, tB as the first frame of a fully formed blastocyst, tEB as the first frame showing expansion of the zona pellucida with enlargement in size. cc2 was calculated as t3-t2. s2 as t4-t3. Two parameters were scored morphologically as binary data; evenness at the two-cell stage was noticed as either even or uneven using software from EmbryoViewer where un-evenness was defined as larger than 20% difference in cell diameter, preferably at the time when both nuclei were visible. The nuclear status at the four-cell stage (multinucleation yes/no) was noted. (For nomenclature, abbreviations and definitions see Table 1).

**Table 1:** Morphokinetic parameters and morphological events included in the time-lapse annotation. The parameters to be annotated were predetermined and agreed upon before the study onset. The parameters are based on recommendations from Ciray *et al.* (2014).

Parameter	Definition	Data collected
**tPNa**	Time at which pronuclei formation can be first identified	Time point
**tPNf**	Time at which both pronuclei have faded	Time point
**tN**	Time from insemination to completion of division of n cells	Time point
**tM**	Time from insemination to formation of a morula, where all cells have compacted and cell membranes are unclear	Time point
**tSB**	Time from insemination to start of blastulation, when the first sign of a cavity formation between two cells is visibly	Time point
**tB**	Time from insemination to formation of a full blastocyst, when the blastocoele cavity fills the embryo with less than 10% increase in its diameter	Time point
**tEB**	Time from insemination to expanded blastocyst, when the blastocyst has increased in diameter with more than 30% and the zona pellucida has started to thin	Time point
**Even-ness**	Even blastomeres at the two-cell stage, less than 20 % difference in size. Preferably at the time when both nuclei are visible	Binary; yes/no
**Multinucleation**	Presence of more than one nuclei in one or more blastomeres at the four cell stage	Binary; yes/no

Using the EmbryoViewer software, the Meseguer selection model (Meseguer *et al*., 2011) was applied to assign each blastocyst with a score, ranging from A+ to E for viable embryos past the fifth cell division. The score was noted for each annotation; hence, each embryo received four scores, two from each embryologist, from two different occasions.

### Evaluation of morphological characteristics:

The same blastocysts were scored using Gardner and Schoolcraft is scoring system (Gardner *et al*., 2000), according to the blastocoel cavity expansion, the number of cells and integrity of both ICM and TD. Twice, each embryologist scored all embryos two months apart, at the time of morphokinetic annotation. The scoring was done using the best available image from the EmbryoScope(tm), between 115-120 HPI. The scoring was done blindly, i.e. the examiner was unaware of the previous score and outcome of transferred embryo.

For statistical purposes, the scored blastocysts were categorized into four classes based on their obtained grade. The blastocysts were classified as belonging to the group Top; blastocysts with an A for ICM and/or TD, to group Fair; blastocysts with B for both ICM and TD, and to group Poor; blastocysts with a C for ICM and/or TD, or Slow; embryos with an expansion grade of 0, 1 or 2 (pre-blastocyst stage embryos).

### Statistics

For morphokinetics, the inter- and intra-observer agreements were evaluated using intra-class correlation coefficient (ICC). ICC provides an estimate that reflects agreement and consistency within assessments. ICC for annotated parameters were calculated using a two-way model with absolute agreement. This gives an ICC single value, which can be interpreted as follows; 0-0.2 indicates poor agreement, 0.3-0.4 indicates fair agreement, 0.5-0.6 indicates moderate agreement, 0.7-0.8 indicates strong agreement and above 0.8 indicates almost perfect agreement (Shrout & Fleiss, 1979).

For morphology, the inter-observer variability was calculated using Fleiss kappa coefficient. Kappa measures agreement between two observers who classified items into categories. If observers are in complete agreement then kappa = 1. If there is no agreement other than what would be expected by chance then kappa= 0. For intermediate values, kappa <0.2 is poor, 0.21-0.40 fair, 0.41-0.60 moderate, 0.61-0.80 good, and 0.8-1.0 very good (Fleiss, 1981). All statistics were calculated in the SPSS.

Implantation rates (IR) were calculated as number of viable fetuses per transferred embryo, as confirmed by ultrasound six weeks after transfer.

## RESULTS

The mean maternal age was 32 years (range 23-40). Of the 99 patient couples, a female cause of infertility was present in 27 cases, male factor in 27 cases, 38 cases of unexplained infertility and 7 couples presented as same sex.

Single fresh embryo transfer of 99 blastocysts to 99 patients resulted in a biochemical pregnancy rate of 53.5 % (53/99) and an ongoing pregnancy rate of 37.3% (37/99). The implantation rate was 37.3% per transferred blastocyst.

### Inter-and intra-observer agreement for morphokinetic parameters

**Figure 1:**
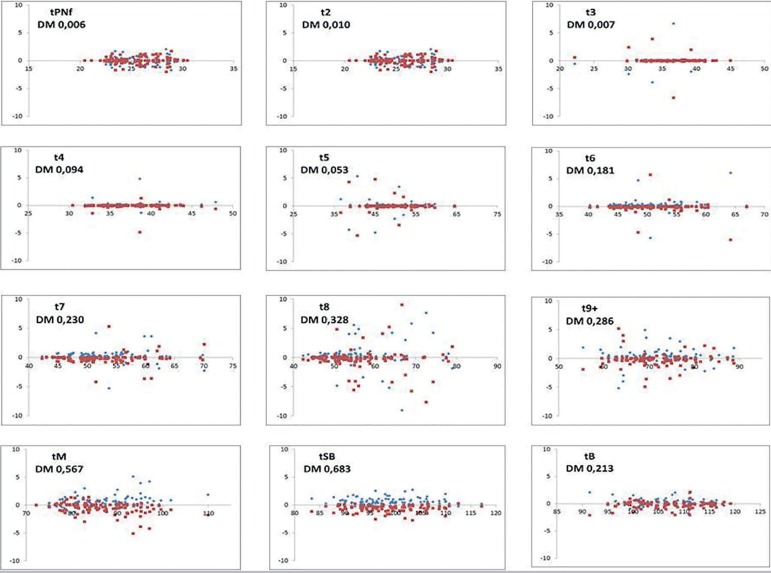
Bland Altman's variability plot. The X-axis shows the mean of the embryologists' morphokinetic annotations, expressed in hours post-insemination. The Y-axis is the difference of the observers' assessment against the mean, expressed in hours. Close clustering to mean equals high agreement. Each plot states name of annotated parameter and calculated difference of mean (DM).

The mean inter-observer agreement between the two embryologists was 0.934, corresponding to an 'almost perfect agreement'. The highest agreement was found for the early events: tPNf and t2, and blastocyst events tSB and tB. The lowest agreement was found for tPNa and t9+, both with values corresponding to 'strong agreement between observers'. The intra-observer agreement, i.e. the consistency of annotating time-lapse images when repeating the measurements was 0.905 (0.753-0.998). Again, lower ICC was found for tPNa and t9+. ICC values are shown in Table 2, and the variability for each parameter is detailed in Figure 1.

**Table 2:** Inter- and intra-observer agreement for 99 transferred embryos with known implantation data. Data expressed as ICC, 95% confidence interval (CI) and number of observations in brackets.

Parameter	Inter-observer			Embryologist 1			Embryologist 2		
	ICC	n	95 % CI	ICC	n	95 % CI	ICC	n	95 % CI
**tPB2**	**0.947**	31	0.892-0.974	**0.944**	31	0.885-0.973	**0.754**	29	0.641-0.876
**tPNa**	**0.753**	50	0.499-0.872	**0.753**	49	0.542-0.865	**0.763**	51	0.620-0.857
**tPNf**	**0.996**	99	0.995-0.998	**0.994**	99	0.991-0.996	**0.996**	99	0.994-0.997
**t2**	**0.997**	99	0.995-0.998	**0.998**	99	0.997-0.999	**0.989**	99	0.984-0.993
**t3**	**0.901**	99	0.856-0.932	**0.964**	99	0.948-0.976	**0.956**	99	0.936-0.971
**t4**	**0.949**	99	0.924-0.965	**0.997**	99	0.996-0.998	**0.990**	99	0.986-0.994
**t5**	**0.955**	99	0.934-0.970	**0.946**	99	0.920-0.963	**0.980**	99	0.971-0.987
**t6**	**0.856**	99	0.793-0.901	**0.901**	99	0.856-0.932	**0.927**	99	0.893-0.951
**t7**	**0.934**	99	0.903-0.955	**0.935**	99	0.905-0.956	**0.905**	99	0.861-0.935
**t8**	**0.810**	99	0.729-0.869	**0.808**	99	0.722-0.868	**0.827**	99	0.753-0.881
**t9+**	**0.753**	95	0.651-0.828	**0.776**	97	0.682-0.844	**0.808**	94	0.725-0.868
**tM**	**0.837**	97	0.760-0.889	**0.866**	96	0.806-0.908	**0.891**	98	0.840-0.926
**tSB**	**0.955**	97	0.862-0.979	**0.961**	97	0.806-0.908	**0.925**	96	0.925-0.960
**tB**	**0.952**	90	0.928-0.968	**0.961**	92	0.942-0.974	**0.923**	90	0.870-0.952
**tEB**	**0.861**	68	0.743-0.921	**0.865**	70	0.771-0.919	**0.861**	68	0.743-0.921
**Median ICC**	**0.934**			**0.911**			**0.899**		
**Mean ICC**	**0.897**			**0.944**			**0.923**		

We further evaluated if high agreement in time-lapse annotations was true also for embryos of all types of quality. 110 embryos from 20 randomly selected patients were annotated on two occasions, two months apart, blinded for previous examinations. Again, both inter- and intra-observer agreement showed strong to almost perfect agreement for all annotated parameters, see Table 3.

**Table 3:** Inter- and intra-observer agreement, for 110 embryos from 20 patients of all types of quality. Data expressed as ICC, 95% confidence interval (CI) and number of observations in brackets.

	Inter-observer			Embryologist 1			Embryologist 2		
Parameter	ICC	n	95 % CI	ICC	n	95 % CI	ICC	n	95 % CI
**tPB2**	**0.868**	26	0.318-0.959	**0.944**	37	0.954-0.988	**0.922**	23	0.809-0.967
**tPNa**	**0.701**	60	0.431-0.836	**0.753**	61	0.793-0.921	**0.772**	63	0.635-0.860
**tPNf**	**0.967**	104	0.951-0.977	**0.994**	109	0.957-0.980	**0.997**	104	0.995-0.998
**t2**	**0.918**	109	0.882-0.943	**0.998**	109	0.953-0.978	**0.975**	110	0.963-0.983
**t3**	**0.927**	108	0.895-0.949	**0.964**	107	0.929-0.966	**0.963**	108	0.946-0.975
**t4**	**0.925**	106	0.891-0.948	**0.997**	106	0.803-0.904	**0.960**	107	0.942-0.973
**t5**	**0.905**	86	0.857-0.937	**0.946**	88	0.850-0.933	**0.942**	87	0.912-0.962
**t6**	**0.880**	80	0.819-0.921	**0.901**	81	0.803-0.917	**0.865**	87	0.801-0.910
**t7**	**0.757**	80	0.646-0.837	**0.935**	78	0.657-0.844	**0.887**	85	0.831-0.925
**t8**	**0.770**	72	0.655-0.849	**0.808**	74	0.689-0.864	**0.855**	76	0.779-0.906
**t9+**	**0.850**	68	0.766-0.905	**0.776**	63	0.900-0.962	**0.775**	72	0.664-0.833
**tM**	**0.937**	59	0.893-0.849	**0.866**	60	0.896-0.961	**0.942**	66	0.906-0.964
**tSB**	**0.907**	48	0.715-0.960	**0.961**	49	0.969-0.990	**0.933**	54	0.883-0.961
**tB**	**0.972**	40	0.948-0.985	**0.961**	40	0.987-0.996	**0.960**	45	0.929-0.978
**tEB**	**0.978**	24	0.946-0.991	**0.865**	26	0.959-0.991	**0.981**	28	0.959-0.991
**Median ICC**	**0.884**	26		**0.911**			**0.915**		
**Mean ICC**	**0.907**	60		**0.944**			**0.942**		

For the observational parameter 'evenness', inter-observer agreement (kappa 0.785) and mean intra-observer agreement (kappa 0.705) were 'very good'. For 'multinucleation' inter-observer agreement was 'fair' (kappa 0.395), but the mean intra-observer agreement was 'very good' (kappa 0.711) I.e., both embryologists were consistent with their own annotation, but not in agreement with each other.

### Inter-and intra-observer agreements for morphokinetic based selection model

We used the Meseguers model (Meseguer *et al*., 2011), strictly as it was published. The agreement was 'very good', with inter-observer agreement in 83 of 99 cases; kappa 0.795, and mean intra-observer agreement with kappa 0.735 (0.650- 0.819). The embryos which scored E in the Meseguer model are, per definition, to be discarded, but since this was a retrospective analysis of transferred embryos with known outcome, grade E embryos were already transferred back to patients. Any discordance between grade E and any other grade means change of fate for the embryo. In eight cases, the decision to transfer/cryopreserve or to discard would change depending on which embryologist performed the annotation of the embryo.

### Inter-observer and intra-observer for morphology

Both inter-observer and intra-observer agreements were 'moderate' for morphology. The two embryologists agreed on the Gardner's Schoolcraft grades (Gardner *et al*., 2000) in 55 of 99 cases, kappa 0.448. Inter-observer agreement was kappa 0.495 (0.464-0.525).

Splitting the morphological assessment of blastocysts into the individual components' expansion grade, TD and ICM showed the highest agreement for TD (kappa 0.706), followed by the expansion grade (kappa 0.670), and the lowest agreement for ICM (kappa 0.542).

**Figure 2:**
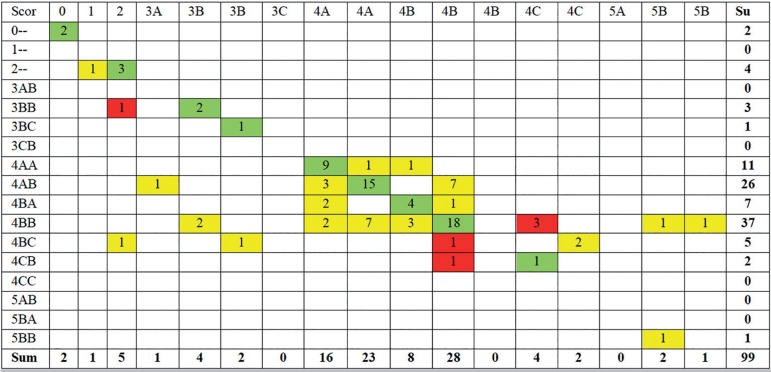
Cross tabulation of embryo scores. This table shows the first morphological scoring of the 99 blastocysts included in the study from the two observers. Scoring was done using the Gardner Schoolcraft's criteria (Gardner *et al*., 2000). In 55 cases, the embryo received the same score from the two embryologists (green cells). This corresponds to a kappa value of 0.448. In 37 cases, the embryos were scored differently by the two embryologists, but with no effect on the fate of the embryo, i.e. transfer/cryopreservation or discard (yellow cells). In six cases, the two embryologists scored the embryos differently, and the different score would have resulted in a clinically relevant change of fate for the embryo, i.e. the embryo would have been discarded by one embryologist and kept for usage by the other (red cells).

The blastocysts graded 3BB or better were transferred and/or cryopreserved in the current clinical IVF program. Exceptions were made to the transfer of lower quality embryos if no better embryos were available. In six cases, the clinical decision would have shifted, i.e. from transfer/cryopreservation to discard or vice versa, depending on which embryologist performed the embryo grading. In the remaining 37 cases, the clinical decision would remain the same, despite different embryo grades. See Figure 2 for details.

### Correlation of morphology and morphokinetics with outcome

**Figure 3:**
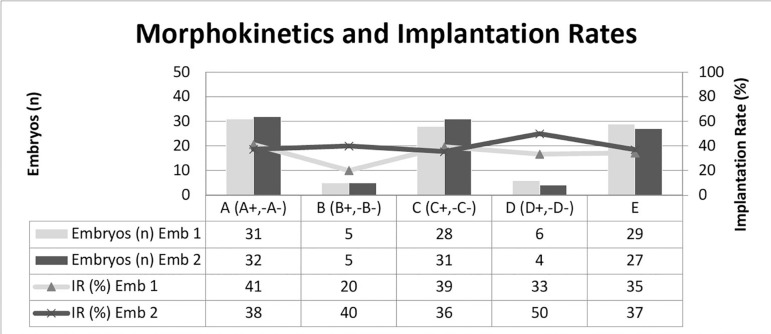
Distribution of embryos into morphokinetic classes based on Meseguer´s selection algorithm (Meseguer *et al.*, 2011). Number of embryos per class, per embryologist on left axis, and corresponding implantation rate per embryologist on right axis.

For morphokinetics, the embryos were ranked into five classes (Meseguer A-E). The distribution of embryos was uneven, with very few embryos assigned as B and/or D. Depending on who scored the embryos, the IR of the classes differed. For embryologist 1 IR were A: 41%, B: 20%, C: 39%, D: 33%, and E: 35%. Corresponding number for embryologist 2 were 38 %, 40%, 36%, 50%, 37%. See Figure 3.

**Figure 4:**
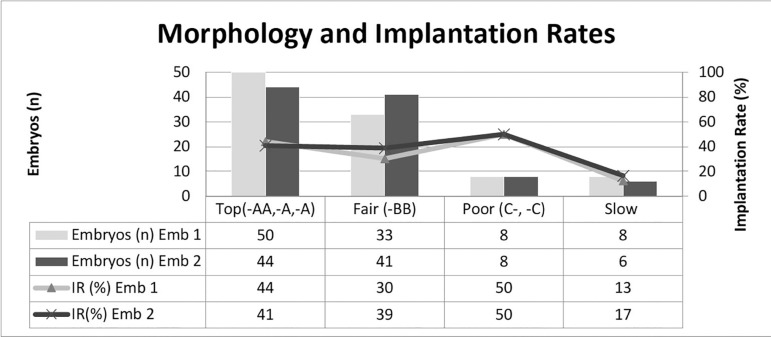
Distribution of embryos into morphological classes based on the Gardner Schoolcraft's criteria (Gardner *et al*., 2000) for the two embryologists, left axis, and corresponding implantation rates (IR) on the right axis.

For morphology, the blastocysts were grouped into four classes (Top, Fair, Poor, Slow). The majority of embryos were classified as Top or Fair. Again, depending on who scored the embryos, the IR for these categories differed. For embryologist 1, the IR was Top: 44%, Fair: 30%, Poor: 50%, and Slow: 12%. For embryologist 2, the corresponding numbers were 41%, 39%, 50% and 17%. See Figure 4. Due to small sample sizes, the significance testing could not be done.

## DISCUSSION

This study shows that the outcome of annotating an embryo using a predetermined list of parameters is independent of who annotates, and when. Previous study by Sundvall *et al*. (2013) reported an inter-observer agreement of 0.81, and intra-observer agreement of 0.85. Our numbers are similar, but slightly higher. In this study, both observers were highly trained embryologists from the same clinic, who used time-lapse cultures for all patients. This may account for the almost perfect agreements. When looking at individually investigated parameters, our results are also in accordance with what has been previously published, with near perfect agreement for early events, slightly lower agreement for events from the three-cell stage to the final cleavage division, and then near perfect agreements for blastocyst events. Even though the EmbryoScope takes pictures in several focal plans, the presence of fragments, and the rapidly changing morphology of cleavage-stage embryos make counting each cell division between day 2 and day 3 the embryo development more challenging.

The static morphological parameter 'evenness at the two-cell stage' showed fair agreement between embryologists and between repeated observations. The use of tools from EmbryoViewer might have improved our results. However, although software tools might aid in measuring diameter and/or circumferences, the embryo might arrange in a manner that makes measuring difficult. This might have accounted for the less than perfect agreement.

For the other static parameter, multinucleation, the agreement was relatively low between embryologists. However, both embryologists annotated in the same manner when repeating their observations two month later. This can be explained by lack of definition of the investigated parameter. Even though we defined multinucleation as the presence of more than one nucleus, we did not define the size of the extra nucleus/nuclei, or duration in time. Since there are many types of multinucleation at the four-cell stage, more pre-annotation strict definitions are needed in order to gain higher accuracy and reproducibility. Once defined properly in the clinic, inter-observer agreement should increase for this parameter as well.

However, our attempt to validate the Meseguer selection model (Meseguer *et al*., 2011) was unsuccessful. The distribution of embryos into subcategories was uneven, and so were the implantation rates. In our hands, the model had a lower performance than in the original publication. There are a number of possible explanations. First, there is increasing, but conflicting evidence that selection algorithms may not be universal. A number of factors may account for that. Stimulation protocols impact morphokinetics (Muñoz *et al.*, 2012; 2013), and the patients in this study were stimulated using both antagonist and agonist protocols, whereas Meseguer *et al*. (2011) used only agonist cycles. The Method of fertilization affects morphokinetics (Kirkegaard *et al*., 2013a; Cruz *et al*., 2013). We included both IVF and ICSI treatments, whereas the Meseguer's study only included ICSI patients. The oxygen concentration has been shown to affect morphokinetics (Kirkegaard *et al*., 2013b). ICSI oocytes were cultured in EmbryoScope directly after injection, and hence cultured in reduced oxygen from the point of fertilization. IVF oocytes were placed in EmbryoScope after fertilization check and exposed to atmospheric oxygen in standard culture incubation from oocyte pick up to fertilization check, approximately 16 hours post insemination. Meseguer *et al*. (2011), on the other hand, used atmospheric oxygen levels throughout the culture period. Indeed, comparing mean cleavage times shows that in our study the investigated embryos cleaved slightly faster compared to the embryos used to design the model. Furthermore, ICSI embryos developed faster when compared to IVF oocytes up until time of morula. See Table 4 for details. The effect of insemination can be eliminated by using tPNf as t=0 instead of the fertilization time.

**Table 4:** Mean annotated time points, expressed as hours post insemination (HPI), for all annotated embryos and for each insemination method. IVF embryos displayed significantly slower kinetics compared to ICSI embryos up until t8.

Parameter	Mean time (HPI) n=99	ICSI (HPI) n=50	IVF (HPI) n=49	∆ IVF-ICSI (HPI)	*p*-value
**tPB2**	3.8±1.2	3.7±1.4	-	-	-
**tPNa**	7.5±1.9	7.5±1.1	7.9±1.0	+ 0.4	n.s.
**tPNf**	23.1±2.6	22.5±2.0	23.6±2.4	+ 1.0	<0.05
**t2**	25.6±2.6	25.1±2.7	26.1±2.5	+ 1.1	<0.05
**t3**	36.5±3.7	35.9±2.6	37.2±3.2	+ 1.4	<0.05
**t4**	37.4±3.6	36.7±4.0	38.1±3.6	+ 1.5	<0.05
**t5**	49.5±5.2	48.6±5.0	50.3±5.2	+ 1.7	<0.05
**t6**	51.1±5.2	50.3±3.3	51.9±4.8	+ 1.7	<0.05
**t7**	53.0±6.2	51.9±5.1	54.1±6.1	+ 2.2	<0.05
**t8**	56.3±9.1	54.7±6.2	57.8±9.0	+ 3.1	<0.05
**t9+**	71.8±7.4	70.4±8.9	73.1±6.7	+ 2.7	n.s.
**tM**	86.5±7.4	86.0±6.9	87.0±9.5	+ 1.0	n.s.
**tSB**	98.6±6.7	98.4±7.0	98.7±6.6	+ 0.3	n.s.
**tB**	106.6±6.2	106.7±6.8	106.4±5.9	- 0.2	n.s.
**tEB**	111.2±5.2	111.0±6.6	111.4±4.9	+ 0.4	n.s.

The use of multinucleation and unevenness as exclusion criteria had a strong impact on our embryo ranking and implantation rates. Per definition, embryos displaying either of these features are scored as 'E'. Looking at all four annotations - two embryologists on two occasions - multinucleation was observed in ~ 10% of the embryos (40/396), resulting in 10 ongoing pregnancies, i.e. IR of 25%. Unevenness was observed in 17% (69/396) of the cases, with 25 ongoing pregnancies; IR 36%. Hence, in our clinic, despite presenting with these features, the embryos still had a high implantation rate.

Since the time of publication, much information regarding multinucleation and development potential has been published. Embryos seem to have a high ability of self-correction, and can develop into euploid blastocysts resulting in live births (Balakier *et al*., 2016). It is possible that the different types of multinucleation present in the cleavage-stage embryos (Meriano *et al*., 2004) have different effects on the developing embryo, and that the type of multinucleation must be further defined in order for multinucleation to be an exclusion criterion in a selection algorithm.

Meseguer *et al*. (2012) replicated their findings in a retrospective multicenter study and in a randomized controlled study (Rubio *et al*., 2014). The latter randomized the patients either to EmbryoScope in reduced oxygen with the hierarchical selection algorithm as embryo selection tool, or conventional culture in atmospheric oxygen, using morphology as an embryo selection tool. Morphokinetics generated higher pregnancy rates; however, the study cannot distinguish between the impact of improved culture condition or improved selection tool. During work with this study, another attempt to externally validate the Meseguer selection algorithm was published (Fréour *et al*., 2015). By applying the model, exactly as it was published, the authors were unable to reproduce the published implantation rates for embryo classes, for neither cleavage-stage embryos nor blastocysts. However, excluding one morphokinetic parameter (cc2), thus creating a simplified model with only five embryo classes, instead of ten, was strongly correlated with implantation, especially for cleavage stage embryos. Essentially, we achieved the same effect of removing cc2, since we combined the ten subgroups into five classes, in order to reduce the number of embryo categories to enable statistical certainty in the analysis.

In 2015, the Meseguer group proposed a new selection algorithm. This model - here named the Basile model - also ranks the embryos into ten categories. After initial removal of morphologically abnormal embryos and embryos displaying multinucleation, unevenness or direct cleavage, in the same manner as the Meseguer model, the morphokinetic parameters t3, cc2 and t5 are utilized. Compared to the Meseguer model used in this study, the parameters are applied in a different order. In their article, the Basile model is validated using 1,620 embryos from several centers that share the same protocols. They report implantation rates that correlate nicely with the embryo scores (Basile *et al*., 2015a,b). The Basile model was externally validated recently, and although the model had potential to identify poor quality embryos and showed significant differences between the best and the poorest score, the sensitivity was reduced (Barrie *et al*., 2017). This study also examined the efficacy of predicting pregnancy from five other morphokinetic models, and concluded that most likely the differences between clinics impacts morphokinetics in such manner that the models cannot be used with the same outcome outside the clinic where the model was built. Most likely, each center needs to gain inspiration from external models, but built and validated their own models using strong, objective, reproducible morphokinetic parameters suitable for their center. The selection algorithms may not be universal, and due to the huge numbers of different factors that differ between clinics, each center should validate their own adequate algorithm for the selection of the best embryo for single transfer using morphokinetics. More evidence to support this comes from another external validation of a blastocyst prediction model (Kirkegaard *et al*., 2014). In this retrospective analysis, the application of a previous published model for blastocyst prediction was somewhat effective to select viable blastocysts with high implantation rates. Nevertheless, as in our study, many embryos that would have been discarded, actually resulted in pregnancies. On the other hand, there seem to be a universal truth regarding the development pattern of an ideal embryo, and a model built using data from multicenter settings would be more likely to fit other clinics, compared to a model built using data only from a single center. An excellent review on morphokinetic theory, selection algorithms and challenges ahead, can be found in Milewski & Ajduk's (2017) recent publication.

Scoring blastocysts using Gardner Schoolcraft criteria (Gardner *et al*., 2000) is standard practice in our clinic. In this study, two experienced embryologists agreed on embryo grade in 55 of 99 cases. In a recent study (Storr *et al*., 2017) they investigated the agreement among Australian embryologists on day-5 blastocysts. When grading the individual components of 100 blastocysts, the agreement was fair to moderate. Their result strongly resembles the results of our study. In both studies, TD (0.397 *vs.* 0.706) and the expansion grade (0.513 *vs.* 0.670) had higher agreement when compared to ICM (0.349 *vs.* 0.542). Our results are slightly better, which might be explained by this being a single-center study, in which the study was set up as a multi-center study. Also in this study, the embryologists had access to 3D video sequences of the blastocyst, in comparison to a 2D image. Both the TD and ICM often require the use of several focal plans to be properly assessed when using EmbryoScope. In six cases, the clinical decision to use or to discard the blastocyst would have changed, depending on who graded the embryo. This highlights the issue of subjectivity when scoring embryos using standard morphology.

It is possible that the strict criteria used for transfer/cryopreservation used in our clinic impacts the embryologist subconsciously. Embryos that are truly a grade C for TD and/or ICM - and therefore are to be discarded - might receive a grade B in order to be used clinically. Comparing the grade given on the day of transfer to the grade given retrospectively in this study shows that six transferred blastocysts indeed received a grade B for ICM and/or TD on the day of transfer, compared to a grade C on in retrospect (data not shown). These subconscious decisions to improve embryo grades have an impact on quality control and benchmarks, and masks possible patient-related issues with embryo development and/or culture conditions.

In conclusion, traditional scoring and selection of embryos using microscopy at predetermined time points has reduced reliability and high inter- and intra-observer variability. The introduction of time-lapse imaging, which captures multifocal images of all embryo development during in vitro culture, has potential to create more objective scoring tools. Most likely, embryo viability is associated with a tight regulated sequence of cellular events that begin at the time of fertilization. Since time lapse provides so much more information about these events, it is fair to assume that more assumptions can be made regarding an embryo's ability to implant or not. This study, the first to compare morphology and morphokinetics on the same set of blastocysts, proves that time lapse annotation is reliable and robust. Further studies are needed to create, implement and validate a selection algorithm for our clinic.
